# Cortical spheroid on perfusable microvascular network in a microfluidic device

**DOI:** 10.1371/journal.pone.0288025

**Published:** 2023-10-19

**Authors:** Teal Russell, Qassim Dirar, Yan Li, Chiwan Chiang, Daniel T. Laskowitz, Yeoheung Yun

**Affiliations:** 1 Fostering Innovation Through Biosystems for Enhanced Scientific Technologies (FIT BEST) Laboratory, Department of Chemical, Biological, and Bio Engineering, College of Engineering, North Carolina A&T State University, Greensboro, NC, United States of America; 2 Chemical & Biomedical Engineering, College of Engineering, Florida A&M University-Florida State University, Tallahassee, FL, United States of America; 3 MIMETAS, BV, DH Oegstgeest, The Netherlands; 4 Department of Neurology, Duke University Medical Center, Durham, NC, United States of America; Eötvös Loránd Research Network Biological Research Centre, HUNGARY

## Abstract

Human induced pluripotent stem cell (hiPSC)-derived brain spheroids can recapitulate the complex cytoarchitecture of the brain, as well as the genetic/epigenetic footprint of human brain development. However, hiPSC-derived 3D models such as spheroid and organoids does not have a perfusable microvascular network, which plays a vital role in maintaining homeostasis *in vivo*. With the critical balance of positive and negative angiogenic modulators, 3D microvascular network can be achieved by angiogenesis. This paper reports on a microfluidic-based three-dimensional, cortical spheroid grafted on the vascular-network. Vascular network was formed by inducing angiogenic sprouting using concentration gradient-driven angiogenic factors in the microfluidic device. We investigate critical factors for angiogenic vascular network formation with spheroid placement, including 1) a PKCα activator, phorbol-12-myristate-13-acetate (PMA); 2) orientation of endothelial cells under perfusion and permeability of vascular network; 3) effect of extracellular matrix (ECM) types and their densities on angiogenesis; and 4) integration with cortical spheroid on vascular network. This paper demonstrates proof of concept for the potential utility of a membrane-free *in vitro* cortical spheroid tissue construct with perfusable microvascular network that can be scaled up to a high throughput platform. It can provide a cost-effective alternative platform to animal testing by modeling brain diseases and disorders, and screening drugs.

## Introduction

Recapitulating brain physiology and pathology *in vitro* is critical for studying disease progression and screening drugs for the treatment of complex neurological diseases such as Alzheimer’s disease (AD) [1–5]. For over a century, two-dimensional (2D) cell culture systems have been used as accepted *in vitro* models to study brain cellular responses. However, 2D models do not reliably recapitulate nor predict brain physiopathology [1, 6, 7]. The current static culturing method, transwell technology [8, 9], uses a flat surface as an insert membrane and a plastic dish. However, this has limited ability to mimic complex multi-tissue interactions with the limited membrane materials. Therefore, there remains a clear need for alternative models to study and better understand human brain physiology and pathology associated with diseases. Recent efforts to develop better *in vitro* brain models include brain spheroids [10, 11], microfluidic-based brain on a chip using primary cells, tissue chip, 3D printing [12], and human induced pluripotent stem cells (HiPSCs)-derived microphysiological systems [11]. HiPSCs have great potential to generate allogeneic or patient-specific cortical cells, tissues, and spheroids/organoids that are physiologically relevant to model neurological diseases. Despite great progress and rapid evolution such as region-specific organoids, vascularized organoids [1, 2, 12–16], and assembloids [1, 17, 18], this area is still in its early stages with several limitations [2]. One important limitation of spheroids/organoids is the absence of a perfusable vasculature network which is a critical component for modeling diseases and screening drugs. In fact, brain is a highly vascularized organ, which provide not only nutrients to meet its high metabolic demands, but that is also removal of toxins that might harm the sensitive neural tissues. Two unique features including the blood brain barrier (BBB) work as a gatekeeper to facilitate the selective trafficking of substances between the blood and the parenchyma and second is with neurovascular coupling to ensure local neuronal activation [19–25]. The loss of vasculature integrity also plays an important role in the onset and progression of not just brain-related diseases such as cerebral amyloid angiopathy, but also in general, blindness, stroke, ischemia, tumor angiogenesis, and arteriosclerosis. Microvascular network can be generated by angiogenesis, which is a complex process where endothelial cells from existing vessels invade as multicellular sprouts to form new vessels with the balance of positive and negative angiogenic modulators such as extracellular matrix and angiogenic factors [19, 20]. To generate microvascular network by angiogenesis needs to carefully investigate and optimize angiogenic factors, fluidic factors such as perfusion/diffusion, extracellular matrix (ECM) types, and their mechanical factors. Typical angiogenic factors includes Vascular endothelial growth factor (VEGF) and fibroblast growth factor (FGF), but PMA as a PKCα activator was not well-studied, which increased the expression of matrix metalloproteinases (MMP), allowing better invasion of endothelial cells for angiogenesis [9, 10].

This paper describes the development of a cortical spheroid grafted on perfusable microvasculature network platform through systematic parameters optimization and characterization including 1) a PKCα activator, phorbol-12-myristate-13-acetate (PMA) to induce angiogenesis; 2) orientation of endothelial cells under perfusion; 3) effect of extracellular matrix (ECM) types and their stiffness on angiogenesis; 4) permeability of vascular network; and 5) integration of cortical spheroid on vascular network. Cortical spheroids are generated from hiPSCs using 1) dual SMAD signaling inhibitors for induction and 2) expansion with cyclopamine (1 μM) and fibroblast growth factor (FGF)-2, and then placed them into the vascularized microfluidic device to generate a cortical spheroid on a vascular bed [24–26]. This microfluidic-based three-dimensional, high throughput, membrane-free vessel, cortical spheroid tissue constructs with vascular network are consisting of 1) a lumenized vascular network and 2) a hiPSC derived cortical spheroid tissue constructs with microvasculature, demonstrating the potential utility for a better understanding of angiogenesis and microvascular formation, for modeling disease mechanisms and progression, for diagnostics, personalized medicine, drug screening, and to possibly reduce the need for animal models.

## Method

### Cell culture: Endothelial

Primary Human umbilical vein endothelial cells (HUVECs) were purchased from Lonza, thawed from liquid nitrogen and were cultured with Endothelial Cell Growth Medium-2 (EGM™-2) and BulletKit™ (Lonza, CC-3162) [10]. We used the cells at P3 till P9. Media were replaced three times a week.

### Undifferentiated human iPSC culture

Human iPSK3 cells (HiPSK3) were derived from human foreskin fibroblasts transfected with plasmid DNA encoding reprogramming factors OCT4, NANOG, SOX2, and LIN28 (kindly provided by Dr. Yan Li with the approval from Stephen Duncan, Medical College of Wisconsin) [11]. As previously reported [11], Human iPSK3 cells were maintained in mTeSR serum-free medium (StemCell Technologies, Inc., Vancouver, Canada) coated with growth factor-reduced Geltrex (Life Technologies). The cells were passaged by Accutase dissociation every 7 days and seeded on six-well plate in the presence of 10 μM Y27632 (Sigma) for the first 24 h.

### Neural differentiation and cortical spheroid formation from human iPSCs in static culture

As previously reported for neural differentiation [11], human iPSK3 cells were seeded into ultra-low attachment plates (Corning Incorporated, Corning, NY) in differentiation medium. Then Y27632 (10 μM) was added during the seeding and removed after 24 h. On day 1, the cells formed embryoid bodies (EBs) and were treated with dual SMAD signaling inhibitors: 10 μM SB431542 (Sigma) and 100 nM LDN193189 (Sigma), hereafter referred to as LDN/SB. After 7 days, the cells were treated with cyclopamine (1 μM) and fibroblast growth factor-2 (10 ng/mL) until day 15 before adding them into vascular network.

### Device preparation and cell culture in microfluidic channels

The microfluidic chip (OrganoPlate Graft, MIMETAS, Netherlands) was used to construct vascularized spheroid tissue. Briefly extracellular matrix (ECM) was prepared; 10×phosphate-bufered saline (PBS) was added to concentrated rat tail collagen I solution (Corning, Collagen I Rat Tail, 10.21 mg/ml) diluted to 5–7 mg/ml with Dulbecco’s Modified Eagle Medium (DMEM) (without supplement), and the pH was adjusted to 7.0–7.4 with 0.5 M (20 mg/ml) NaOH. Prepared collagen solution was injected into gel inlet and polymerized in incubator for 15 minutes. Suspended endothelial cells in EGM-2 medium was seeded into both perfusion inlet channels and place them in incubator to allow cells to attach. Additional medium (50 μL) was loaded into the inlet well and the plate was placed on an interval rocker (MIMETAS, The Netherlands), allowing bi-directional flow for perfusion. Medium was refreshed every 2 days. Tissue chip was maintained in EGM-2 medium (changing every 2 days) in a rocker (OrganoFlow, Mimetas, USA) until it formed tubular structure. As a next step, angiogenic cocktail with VEGF (37.5 ng/mL, Preprotech, #100–20), bFGF (37.5 ng/mL, Peprotech, #G00832 100-18B), Monocyte chemoattractant protein-1 (MCP-1) (37.5 ng/mL), Hepatocyte growth factor (HGF) (37.5 ng/mL), Sphingosine-1-phosphate (S1P) (Sigma,#G00918, 250 nM in 5% 1 M HCl, 95% DMSO), and PMA (37.5 ng/mL, Sigma, # P1585) suspended in EGM-2 was added to gel chamber only to generate concentration gradient. Medium was changed every 2 or 3 days. To assess the effects of PMA exposure on sprouting, we prepared cocktail with and without PMA in EGM-2 medium. As sprouting capillary vasculature are fully developed to the spheroid inlet, we placed the cortical spheroid and vascularized-tissue was recorded every 2d by performing phase-contrast microscopy.

#### Microfluidic concentration gradient chip

**Fig 1:**
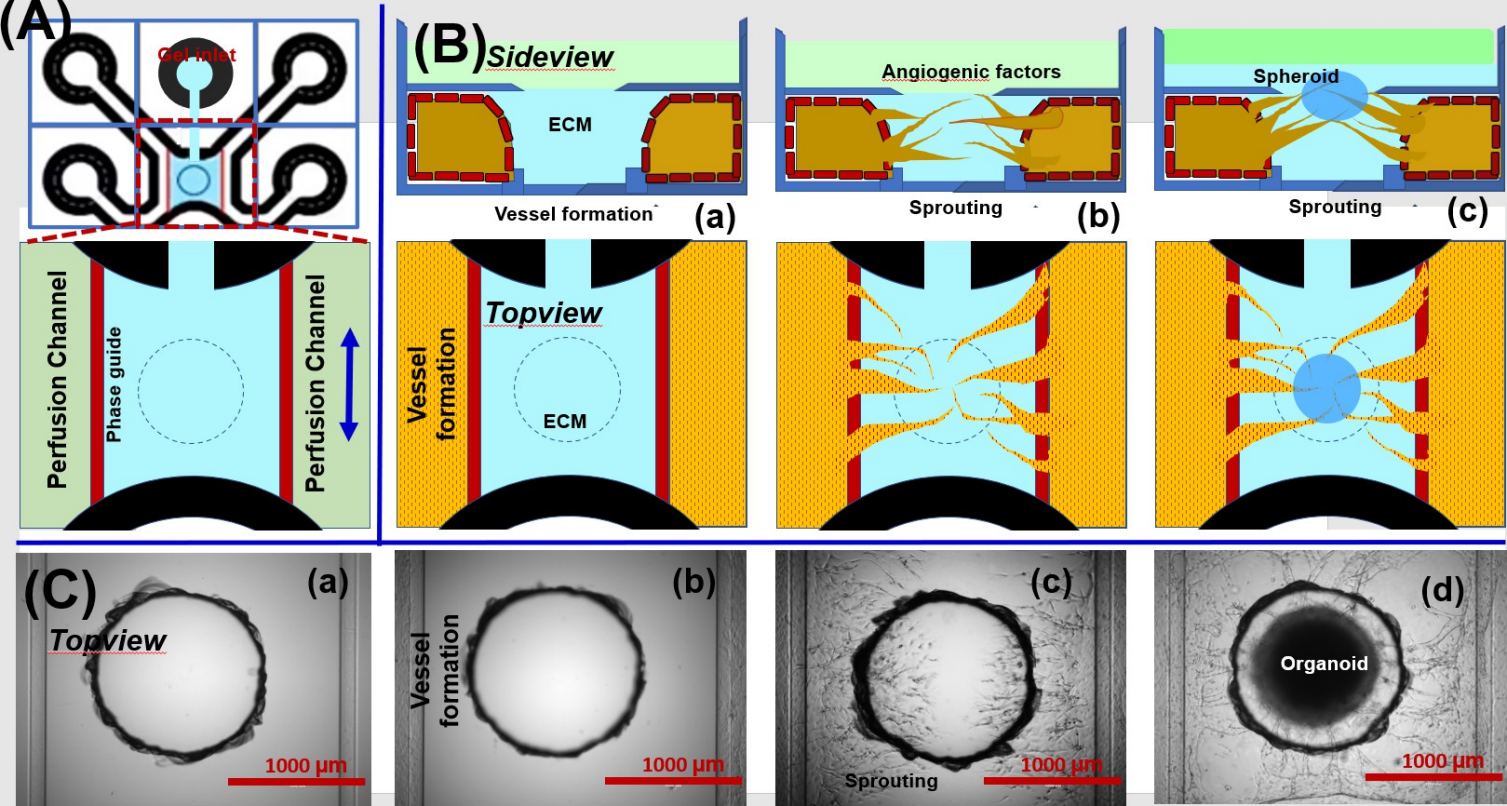
Reproducible 3D-vascularized spheroid tissue construction process of high content/high throughput platform. **(A)** structure of OrganoPlate Graft in 384 well plate format, **(B)** experimental procedure for constructing 3D-vascularized spheroid tissue, and **(C)** represented images of each brain tissue construction process step; (a) collagen-I loading, (b) endothelial cell seeding in the perfusion channels and vessels formation under flow, (c) vascular network formation using an angiogenic cocktail, and (d) cortical spheroid placement and maturation onto the pre-formed vascular network.

**Fig 1(A)** shows the process used to construct the cortical spheroid grafted vascular bed using the OrganoPlate Graft. This microfluidic platform has 64 independent tissue culture chips per well-plate. Every unit consists of three channels and one grafting chamber. Center channel is used to pattern an extracellular matrix (‘gel channel’), two adjacent channels (‘perfusion channels’) are used to generate endothelial barrier against gel and further angiogenesis, and one open grafting chamber is used to insert spheroid. The channels are separated by PhaseGuides [27], small ridges that function as capillary pressure barriers, which enable patterning of cells and gel without the use of physical membranes [27]. Every channel has one inlet and one outlet, which connect the channels with the wells in the microtiter plate (medium reservoir). Hydrogel is filled to the center to the grafting chamber, which have a high concentration of angiogenic cocktail (source), generating concentration gradient from this open grafting chamber to the perfusion channels (sink). To stabilize the gradient over time, the device was placed on a rocker platform to perfuse both perfusion channels continuously and simultaneously. It was previously reported that a gradient is present after 6 days [28]. We changed a growth factor and media were replaced at every 2–3 days to maintain concentration gradient. Microvessels cultured against patterned collagen-1 hydrogel: Collagen-I hydrogel was loaded using capillary pressure barriers which enable separation of gel and fluid phases and form a membrane-free substrate for endothelial cell attachment. After collage-I polymerization, suspended endothelial cells was seeded against the collagen-I matrix of the two perfusion channels. Following stable attachment on the collagen matrix, the plate was kept on a perfusion rocker which provided fluidic flow for 2 or 3 days (estimate shear stress; ~1.0–1.5 dyne/cm^2^), forming vessels under gravity-driven flow using a slope controllable rocker (**Fig 1(B-A)**). Endothelial cells formed a confluent monolayer after 3 days of culture, and then formed a tube-like micro-vessel structure [10]. The apical side of the vessel (the lumen) can be accessed through the perfusion channel, while the gel forms the basal side of the tube.

#### Sprouted micro-vascular network (Angiogenesis)

After reaching confluency, the microvessels show a stable morphology of a single monolayer against the collagen I hydrogel. Vascular network with newly sprouted vessels was developed by placing an angiogenic cocktail on top graft inlet of the collagen hydrogel, which forms a gradient towards the perfusion channels, thereby attracting angiogenic sprouts (**Fig 1(B-B)**). The angiogenic cocktail we investigated include VEGF (37.5 μg/mL), FGF-2 (10 μg/mL), MCP-1 (37.5 μg/mL), HGF (37.5 μg/mL), S1P (250 nM), and PMA (37.5 μg/mL) suspended in EGM-2 [2, 3, 28, 29]. This induced the formation of tip and stalk cells after respectively 1 and 2 days. We quantified the angiogenesis including tip/stalk cell formation, the presence of filopodia and lumen formation and directional growth towards the gradient [30–32].

#### Spheroid grafting

We selected the spheroid (0.5 to 0.7 mm diameter) after the treatment with cyclopamine (1 μM) and FGF-2 for 7 days [11]. Generated cortical spheroid was inserted on the grafting chamber of OrganoPlate Graft and further additional ECM such as collagen or Matrigel was added to fully encapsulate the spheroid. From this point, we switch to the medium supplemented with BDNF for spheroid maturation (not angiogenic cocktail anymore) for more than 7 days (**Fig 1**). **Fig 1C** shows the represented images of each brain tissue construction step; (a) loading of collagen hydrogel, (b) endothelial seeding in the perfusion channels to form vessels under fluidic flow, (c) vascular network formation using an angiogenic cocktail to stimulate sprouting, and (d) spheroid placement onto the pre-formed vascular network.

#### Immunocytochemistry

The cell culture medium was aspirated and 75 μL of 4% paraformaldehyde (PFA) in phosphate-buffered saline (J61899, Alfa Aesar, USA) was added to all the perfusion inlet and outlet wells [10]. The device was placed on a rocker to induce flow and incubated overnight at 4°C. After fixation, the PFA was aspirated from the wells and the microfluidic chips were washed three times with 0.3% Triton X-100 in PBS in all the perfusion inlets and outlets, followed by a permeabilization step of 1% Triton-X100 (VWR 28817295). Nuclei were stained using 1:2000 Hoechst 33258 (H3569, Life Technologies, USA). Tissue construct was incubated with primary antibodies for overnight; 10 μg/mL αβ3-tubulin, AF488 (Invitrogen, 53-4510-82), 0.36 μg/mL αCD31, Rabbit (Invitrogen, PA5-16301), 5 μg/mL αPECAM1 (Millipore Sigma, WH0005175M1), 10 μg/mL αVE-Cadherin, Rabbit (Sigma-Aldrich, V1514), and 5 μg/mL αZO- 1, Mouse, AF488 (Invitrogen, 339188). Secondary antibodies with blocking solution are further incubated; 2 μg/mL αMouse, AF568 (Invitrogen, A-11004), 4 μg/mL αRabbit, AF568 (Invitrogen, A-11036), and 2 μg/mL, αRabbit AF488 (Invitrogen, A-11008). Image data was acquired and processed using confocal microscope (ZEISS Multiphoton LSM 710) and ZEN software. Imaging depth was set at 16 bits, binning at 1, and imaging resolution at 2048 × 2048. Autofocus was set at a 120 μm offset from channel bottom.

#### Immunohistochemistry

Tissue construct was extracted from the OrganoPlate Graft. Tissues were fixed in 4% paraformaldehyde overnight at 4°C followed by washing in PBS 3 times for 5 min. Tissues were allowed to sink in 30% sucrose overnight and then embedded in OCT, flash frozen, and cryosectioning at 14 μm. Section were blocked and permeabilized in 0.2% Triton-X in PBS. Sections were then incubated with primary antibodies in 0.1% Tween-20, 10% normal donkey serum at the following dilutions: 0.3 μg/mL βIII-tubulin, Chicken (Millipore Sigma, AB9354), 10 μg/mL αTBR1, Mouse (Proteintech, 66564-1-Ig), 2 μg/mL αSATB2, Rabbit (Invitrogen, PA5-83092), 2 μg/mL αN-Cadherin, Rabbit (Invitrogen, PA5-85495), and 4 μg/mL αβ1-Integrin, Mouse, AF647 (Santa Cruz Biotech, sc-9970). Nuclei were stained using 1:2000 Hoechst 33258 (H3569, Life Technologies, USA). Secondary antibodies are further incubated; 3 μg/mL αMouse, AFP488 (Invitrogen, A32766), 3 μg/mL αRabbit, AF568 (Invitrogen, A-11036), 5 μg/mL αRabbit, AF488 (Invitrogen, A-11008), 5 μg/mL αChicken, AF647 (Invitrogen, A-21449), and 5 μg/mL αChicken, CF594 (MilliporeSigma, SAB4600094).

#### Sprout permeability visualization

As previously reported [10, 28], 50 μL of a 70 kDa FITC-Dextran (Sigma #53471) solution (0.5 mg/mL in EGM-2 culture media) was added to the perfusion inlet well at day 4 and day 9 after stimulation, and time lapse images were acquired at 1 min intervals using a confocal microscope.

#### Sprouting quantification

The sprouting was quantified by counting the number of vessels and measuring their length using Fiji (Image J). The vessels length was measured by tracing the vessel from the edge of the perfusion channels (on both sides) to its tip in the ECM.

#### Quantification of HUVEC orientation

The orientation of HUVEC cells was calculated using the directionality plugin in Fiji. The directionality plugin produces a histogram of the orientation of structures present in the image. The orientation histogram was generated using the Fourier method consisting of 10 bins starting at 0° and ending at 180°. The histogram was then converted into a polar histogram and plotted on polar coordinates.

#### Statistics

Statistical analysis was performed using GraphPad PRISM 8 (CA, USA). Data are represented as mean ± standard deviation (SD) and analyzed using unpaired two-tailed student t-test, with α = 0.05 and statistical significance accepted at p < 0.05.

## Results

### Perfusable microvascular network generation

**Fig 2:**
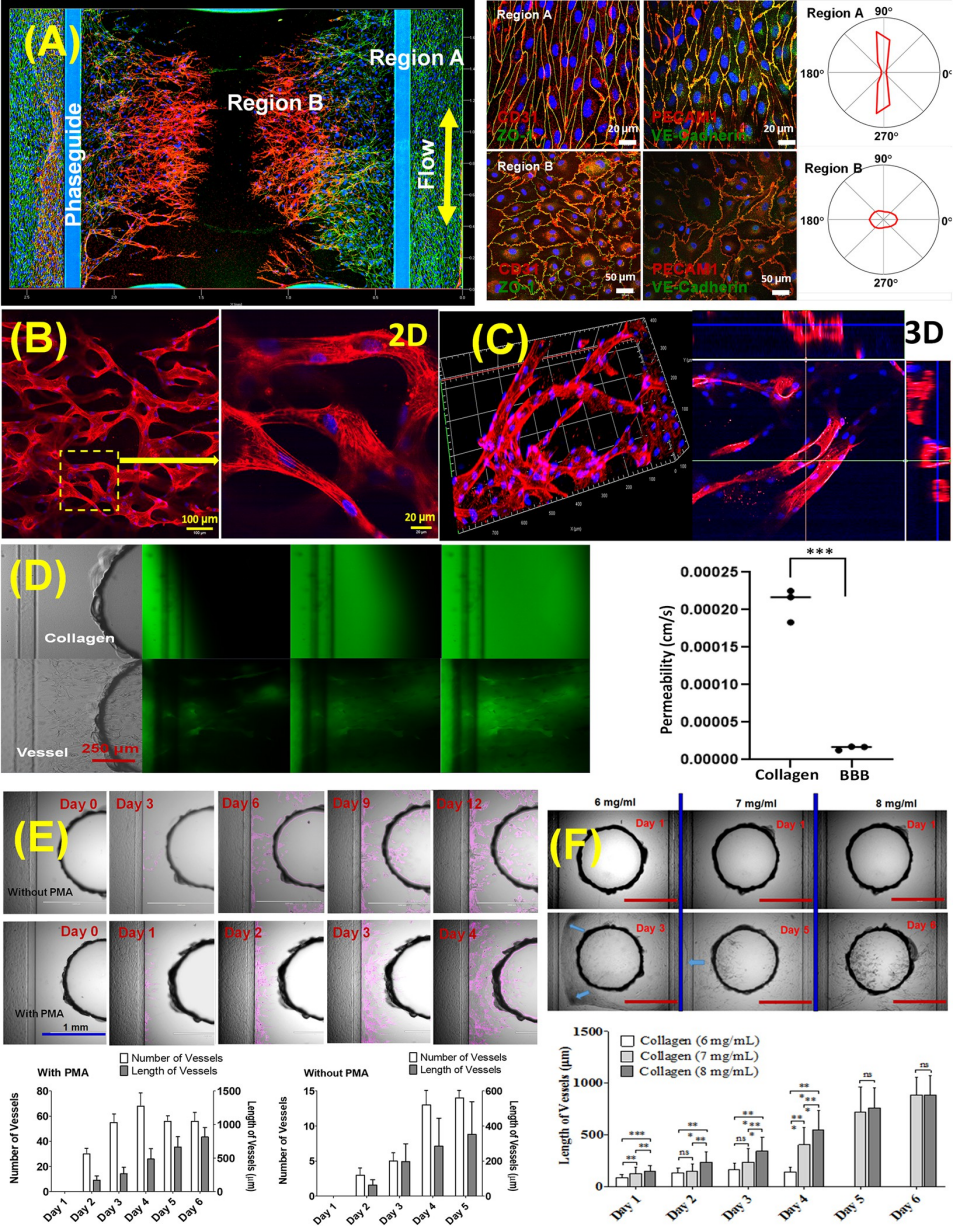
Angiogenic-derived microvascular network. **(A)** Microvasculature morphology in the OrganoGraft: **Region A** (perfusion channel) and **Region B** (new angiogenic vasculature), effects of shear stress on endothelial cell alignment, and polar histogram quantification of cell alignment with respect to bidirectional flow using a rocker (**Right Figures**). **(B-C)** Representative images of vascular network acquired from F-actin staining **(B)** 2D and **(C)** 3D images with cross-section view of vascular network. **(D)** Permeability of vascular network acquired as time lapsed images after the addition of the FITC-dextran of collagen-I matrix only (control) and vascular network with collagen-I matrix (pictures were taken at every 2 minutes). (Significance was calculated using unpaired two-tailed student t-test, *** P<0.001). **(E)** Represented angiogenesis of endothelial cells and quantification of angiogenesis in number and length of micro-vasculature with and without PMA in angiogenic cocktail (pink color shows sprouted vessels using Image J, n = 3). **(F)** Represented angiogenesis with collagen density of 6 mg/mL, 7 mg/mL, and 8 mg/mL. Microvessels were pre-formed for 3 days in perfusion environment at both perfusion channels and induced gradient driven sprouting (Day 0) using angiogenesis cocktail of containing with VEGF (37.5 μg/mL), FGF-2 (10 μg/mL), MCP-1 (37.5 μg/mL), HGF (37.5 μg/mL), and S1P (250 nM), without and with 2 ng/mL PMA addition. (Significance was calculated using unpaired two-tailed student t-test, * P<0.1, ** P<0.01, *** P<0.001).

Microvascular network was formed by inducing angiogenesis (**Fig 2**). Microvessels were grown on both perfusion channels to form a confluent monolayer of endothelial cells and maintained in the rocker for 2–3 days to form a tube-like structure. The device was then stimulated with an angiogenic cocktail containing VEGF (37.5 μg/mL), FGF-2 (10 μg/mL), MCP-1 (37.5 μg/mL), HGF (37.5 μg/mL), S1P (250 nM), and PMA (37.5 μg/mL) to create vascular network. We investigated the morphology of newly sprouted vascular network under perfusion. **Fig 2(A)** shows the stained images of tight junction (ZO-1, green) and platelet endothelial cell adhesion molecule- 1 (PECAM-1; also known as CD31, red) in the microfluidic device. As the device was kept on the perfusion rocker at a 7-degree angle, which generates shear stress from 0.05 Pa to 0.1 Pa [10, 27]. This perfusion changed the orientation of endothelial cells in a flow rate-dependent manner toward the direction of flow within the perfusion channels (**Fig 2(A), Region A**). Prior reports have also demonstrated that exposure to shear stress reoriented the cells in the direction of the medium flow and further accelerated tubular structure formation [33]. On the other hand, newly sprouted vasculature (**Region B**) is maturated under diffusion in the collagen-I matrix, which are more randomly oriented and larger size of endothelial cells are observed than endothelial cell in perfusion channel. The results show that the junction proteins, ZO-1 are strongly localized at the cell junctions and PECAM-1 are expressed on cell junctions as well as the cell adhesion to collagen matrix. Expression of cell junction on the microvessel (**Region A**) was higher than the expression on the new sprouted vasculature (**Region B**). Quantitative results of endothelial cells orientation using polar histogram plot shows the difference between **Region A** and **Region B**. F-actin staining results show that sprouted vascular network induced by angiogenesis is well-connected from both perfusion channels to the graft chamber (anastomosis) for trafficking oxygen, nutrients, growth factors and waste (**Fig 2(B) and 2(C))**. Confocal images in **Fig 2(C)** show that the angiogenic sprouts have a clear lumen structure and appear circular shape in a cross-sectional view. Even though we did not report, we observed that, with prolonged exposure to angiogenesis cocktail, connected channels under perfusion maintained stable lumens and formed a functional barrier. Endothelial permeability was determined by adding 10 μM FITC-dextran (MW: 70 kDa) to the perfusion channel after constructing vascular network. Fluorescent images were acquired as time lapsed images after the addition of the FITC-dextran solution. As shown in **Fig 2(D),** in the absence of vascular network (collagen I matrix only), FITC-dextran was diffused from left perfusion channel to right side’s center uniformly (average permeability was 0.0002083 cm/s). In the case of sprouted vascular network (**Fig 2(D)**), FITC dextran was firstly transported though sprouted vessels due to well-connected vascular network (average permeability was 0.0000157 cm/s). We further investigate the effect of PMA (a PKCα activator), which increase the invasion activity by the expression of matrix metalloproteinases (MMP) -2 and -9. As shown in **Fig 2E**, in the presence of PMA, the tip-cells immediately invade the collagen-1 scaffold and kept visible 24 h after exposure. The first lumens are visible 48 h after cocktail exposure with PMA while the tip cells have migrated further in the direction of the gradient. We observed that sprouted angiogenic vasculature network is formed to the center graft chamber within 5 days. As shown in **Fig 2(E)**, with PMA addition, both number and length of sprouted microvasculture are rapidly increased from 10 (± 3) without PMA to 60 (± 10) vessels and increased from 380 (± 150) μm without PMA to 800 μm (± 200) vessel length with PMA., which is critical for angiogenesis. We investigated the effect of collagen-I matrix density on sprouted vascular network formation (**Fig 2(F)**). We varied densities of collagen, 6, 7 and 8 mg/mL and quantified sprouted angiogenesis in number and length of micro-vasculature. In the case of 6 mg/mL collagen, as collagen could not hold sprouting vessel and collapsed at day 3 or 4 (green arrows in **Fig 2(F)**). On the other hand, 8 mg/mL sample provided environment to induce angiogenesis of endothelial cell and maintained the structure integrity with sprouted vascular network. We could not test more than 8 mg/mL due to limitation of pressure barrier of meniscus pinning.

### Cortical spheroids in the microfluidic device

**Fig 3:**
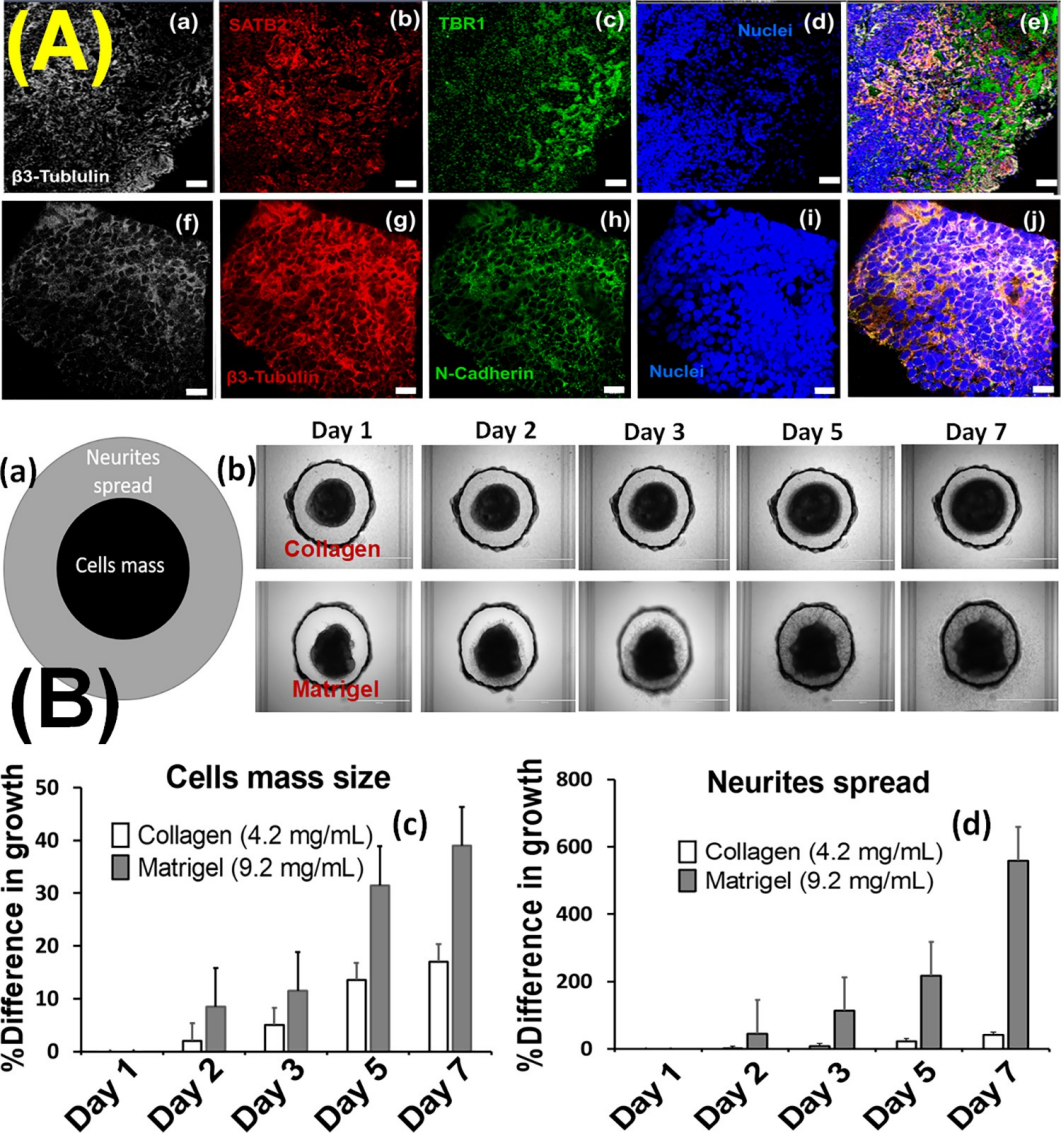
**(A)** Representative fluorescent images of cortical neural markers and adhesion markers expressed in derived cortical spheroids. (a) White: beta III-tubulin, (b) Red: SATB2, (c) Green: TBR1, (d) Blue: Nuclei (Hoechst), (f) White: β1-integrin, (g) Red: β3- tubulin, (h) Green: N-cadherin, (i) Blue: Nuclei (Hoechst). Scale Bar: 50 μm. **(B)** (a) Schematic diagram of cell mass and neurite spared, (b) represented images of cortical spheroid in Collagen and Matrigel as well as (c) quantitative data of spheroid diameter and (d) neurite outgrowth of cortical spheroid in Collagen and Matrigel in days.

Cortical spheroids were generated by treating iPSK3 with ROCK inhibitor followed by dual SMAD signaling inhibitors of LDN/SB. Expansion of spheroids was further achieved by cyclopamine and FGF-2. **Fig 3(A)** shows the representative fluorescent images of cortical neural markers (TBR1 and SATB2) and adhesion markers (β1-integrin and N-cadherin) expressed in cortical spheroids before adding them into vascular network. As shown in **Fig 3(A)**, fluorescence images from IHC show the expression of TBR1 (deep cortical layer VI) and SATB2 (superficial cortical marker II-IV). The expression of beta III-tubulin was distributed throughout the spheroids. IHC results revealed that the spheroids were solid with small round cells evenly distributed and did not see any necrotic core found in the spheroid [11, 24]. In order to study the migration of neurons from cortical spheroids in ECMs of collagen and Matrigel, we embedded these spheroids and studied how neurons are migrating in days *in vitro*. **Fig 3(B)** shows the represented images of spheroid expansion with days and their quantitative data for neurite outgrowth and cell mass. We observed the increase of diameter on both spheroid mass and neurites outgrowth. Spheroid in Matrigel have higher expansion rate than spheroid in collagen.

### Cortical spheroid on the perfusable vascular network

**Fig 4:**
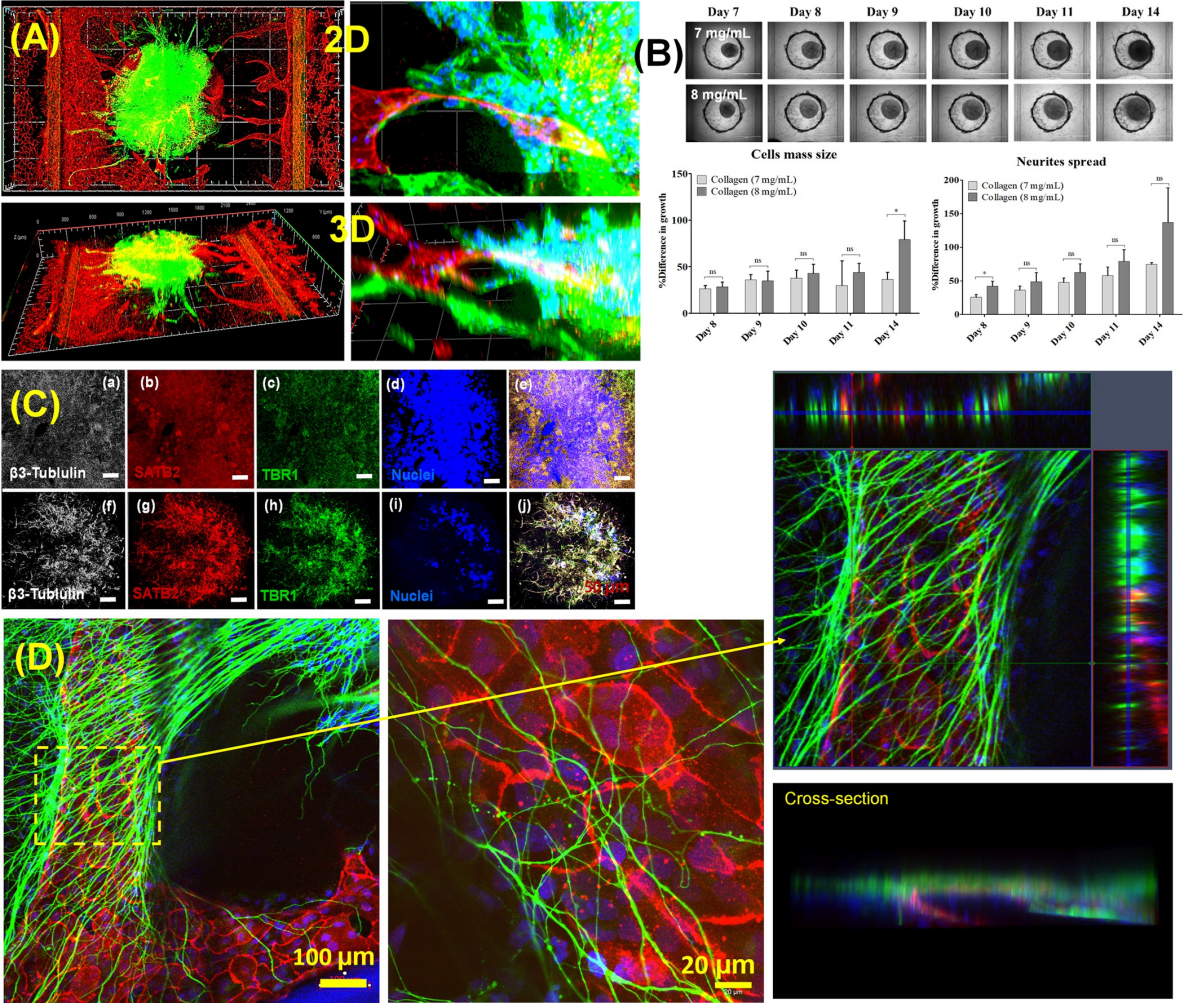
Cortical spheroid on vascular network. **(A)** 3D and 2D confocal images of spheroid on vascular network (Red (CD31) is microvessel and green (beta III tubulin) is cortical spheroid), **(B)** represented images of cortical spheroid growth and spreading on vascular network and quantitative data for spheroid diameter and neurite outgrowth of cortical spheroid in Collagen (7 mg/mL) and (8 mg/mL), **(C)** fluorescent images of cortical neural markers from the middle (upper one) and outer (lower one) regions after extracted from vascular network. White: beta III-tubulin, Red: SATB2, Green: TBR1, and Blue: Nuclei (Hoechst). Scale Bar: 50 μm, and **(D)** 2-Week culture of cortical spheroid on vascular network and cross-section views of neurites-wrapped vascular network (green is beta III tubulin and red is CD31).

After the vascular network was fully developed to the graft chamber, cortical spheroid was placed to the graft chamber and additional collagen I or Matrigel was added to fully maturate the cortical spheroid. We changed the EGM-2 medium to DMEM medium with BDNF for spheroid formation (no angiogenesis cocktail was added). Even though we did not supply the angiogenesis factors, we observed that sprouted micro vasculature maintained vascular network with cortical spheroids. BNDF based medium continues to preserve cortical spheroid maturation. Even though diffusion of antibodies hinders the staining the spheroids [11], we still see the overall morphology of spheroid on the vascular network (**Fig 4**). Side and top view in **Fig 4(A)** show the how sprouted vasculature are networked to the spheroid (yellow is overlapped area). Magnified view in **Fig 4(A)** demonstrates integration of capillary vessels and neurons. We observed that neurons’ migration along with the capillary vessel and some capillary vessel integrated with spheroid.

We investigated the effect of collagen-I matrix density on spheroid expansion of spheroid on the vascular network in terms of spheroid diameter and neurite outgrowth in Collagen of 7 mg/mL and 8 mg/mL (**Fig 4(B)**). We did not observe the significant difference of cell mass size and neurite spread in spheroid between Collagen 7 mg/mL and 8 mg/mL. We extracted spheroid from vascular network and characterized the cortical markers from the middle (upper row in **Fig 4(C)**) and outer (lower row in **Fig 4(C)**) regions of spheroids. IHC fluorescent images show cortical neural markers of TBR1 and SATB2 expressed in iPSK3 neural spheroid. **Fig 4(D)** shows the 3D confocal images of vascularized cortical spheroid for 2 weeks incubation (Dimension: 800 μm x 800 μm x 160 μm with 35 slices). Enlarged 3D images with cross-section views show that neurites from cortical spheroid were further expanded to vasculature and wrapped outside of vasculature (3D movie is also attached at S1 Video). High resolution image in **Fig 4(D)** shows the neurites are integrated with basal membrane of endothelial cells.

## Discussion

In the present study, we constructed cortical spheroids grafted on the vascular bed in ECM hydrogel by combining 3D angiogenesis and spheroid models. This microfluidic platform provides an *in vitro* grafting of cortical spheroid on the dynamic, membrane-free vascular network bed in high throughput format. This represents a combination of features enhancing both brain physiological relevance and potential use for throughput not previously achieved with *in vitro* models of the cortical spheroid on vascular bed. To demonstrate the utility of this model, we evaluated the vascular morphology, vascular network function, and further cortical spheroid interaction. This passive system does not require complex pumps and tubing, allowing for multiple high throughput assays [28]. Even though it is known that unidirectional flow is important for remodeling during angiogenesis [9], we observed that vessels under bi-directional perfusion are aligned with flow direction and formed barrier function such as tight junction. This microfluidic chip platform generated concentration gradient for angiogenesis cocktail, which 1) induces differentiation of endothelial tip cells, 2) grows out from a pre-existing vascular network into the extracellular matrix, 3) followed by stalk cells to the tip cell to form a vessel lumen, and 4) acquire phalanx cell (quiescent EC phenotype) to maintain new vessel [30, 34–37]. Angiogenic vascular network demonstrated clear lumen structure formation as a circular shape which maintain barrier integrity in collagen matrix. After initial sprouting, angiogenic vessels went through pruning, anastomosis and widening processes [34]. We found that medium perfusion through anastomosis is critical to maintain stable sprouted vessels.

We observed that PMA is a strong inducer of sprouting angiogenesis [28, 33, 38]. PMA disrupts the integrity of the endothelial cell layer and upregulates the synthesis of proteinases, such as matrix metalloproteinases, that are essential for cell invasion in the early stages of angiogenesis [30, 39]. PMA significantly accelerated migration, as well as vascular network formation and stabilization. Endothelial cells in sprouted vessel against collagen I matrix exhibited an increased spread area, higher circularity, and a larger cytoplasm compared to cells under perfusion. PMA triggered collective cell migration in the form of directional, long, and multicellular sprouts into the collagen matrix [28, 33, 40]. As previously reported by V. van Duinen [28], connected channels maintained stable luminal structure while disconnected vessels are collapses, followed by regression of the angiogenic sprouts (pruning process) [27]. Connected channels under perfusion maintained stable lumens and formed a functional barrier. Permeability of vascular network with FITC dextran (MW: 70 kDa) show that vascular network is well-connected, but barrier is a little leaky probably due to early stage of new vessel’s maturation and measurement at room temperature. Vascular permeability in our model was 1.57 x 10^−5^ cm/s which is still higher than rat model with 10 kDa dextran (3.1 × 10^−7^ cm/s) and 1.4–1.9 × 10^−7^ cm/s in rat and mouse with 40 kDa dextran [20].

HiPSC-derived cortical spheroids grafted on the vascular bed was generated. We separately expanded and inserted human iPSC spheroid in this microfluidic platform, which allows the study of vasculature and spheroid interactions that are critical for recapitulating human neurological disease. We observed that some neurons and their neurites from cortical spheroid migrated to vasculature and vascular network bed continuously engaged with cortical spheroid. In particular, we observed physical attachment of neurites to BBB allowing direct interaction. As we reported previously [24–26], we did not observe any necrotic core in this specific culture condition, probably because of the relative smaller size of spheroid with a short-term culture time and iPSK3 cells we used in this paper did not form multiple cortex layers.

Although further studies are necessary to investigate certain aspects of this technology, such as the migration of vasculature inside the cortical spheroid and the impact of cortical neurons on the endothelial blood-brain barrier, this study provides a proof-of-concept for *in vitro* spheroid grafting on a vascular network bed in a high-throughput manner. This technology has the potential to be used to model brain diseases [41] and screen compounds in a more cost-effective and ethical manner than animal testing. Personalized medicine could also benefit from this technology by enabling patient-specific drug screening and reducing the risk of adverse effects. However, more long-term studies and novel characterization methods will be necessary to fully realize the potential of this technology.

## Conclusion

This paper presents a proof-of-principle study demonstrating the integration of vascular networks with spheroids, specifically a vascularized human iPSC-derived cortical spheroid. The study utilized a perfused micro-vessel with an endothelial layer, a vascular network induced by a concentration gradient-driven angiogenic cocktail, and a spheroid. This technology enables real-time study of interactions between neurons and the vascular system and has potential applications in personalized medicine, drug screening, and modeling brain diseases. However, the limitations of using HUVEC for endothelial cells suggest that the use of iPSC-derived brain endothelial cells and further addition of astrocyte and pericyte can better recapitulate the blood-brain barrier, leading to more accurate disease modeling in the future. Overall, this study presents a promising technology that has the potential to advance our understanding of the brain’s complex biology and to accelerate drug discovery and development.

## Supporting information

S1 File (XLSX)

S1 Video (AVI)
